# A novel contribution of *spvB* to pathogenesis of *Salmonella* Typhimurium by inhibiting autophagy in host cells

**DOI:** 10.18632/oncotarget.6989

**Published:** 2016-01-22

**Authors:** Yuanyuan Chu, Song Gao, Ting Wang, Jing Yan, Guangmei Xu, Yuanyuan Li, Hua Niu, Rui Huang, Shuyan Wu

**Affiliations:** ^1^ Department of Microbiology, Medical College of Soochow University, Suzhou, P. R. China

**Keywords:** Salmonella, spvB, autophagy, autophagic flux

## Abstract

*Salmonella* plasmid virulence genes (*spv*) are highly conserved in strains of clinically important *Salmonella* serovars. It is essential for *Salmonella* plasmid-correlated virulence, although the exact mechanism remains to be elucidated. Autophagy has been reported to play an important role in host immune responses limiting *Salmonella* infection. Our previous studies demonstrated that *Salmonella* conjugative plasmid harboring *spv* genes could enhance bacterial cytotoxicity by inhibiting autophagy. In the present study, we investigated whether *spvB*, which is one of the most important constituents of *spv* ORF could intervene in autophagy pathway. Murine macrophage-like cells J774A.1, human epithelial HeLa cells, and BALB/c mice infected with *Salmonella* Typhimurium wild type, mutant and complementary strains (carrying or free *spvB* or complemented only with ADP-ribosyltransferase activity of SpvB) were used *in vitro* and *in vivo* assay, respectively. To further explore the molecular mechanisms, both SpvB ectopic eukaryotic expression system and cells deficient in essential autophagy components by siRNA were generated. Results indicated that *spvB* could suppress autophagosome formation through its function in depolymerizing actin, and aggravate inflammatory injury of the host in response to *S.* Typhimurium infection. Our studies demonstrated virulence of *spvB* involving in inhibition of autophagic flux for the first time, which could provide novel insights into *Salmonella* pathogenesis, and have potential application to develop new antibacterial strategies for Salmonellosis.

## INTRODUCTION

*Salmonella enterica* is associated with 93.8 million cases of gastroenteritis and 21.7 million cases of systemic typhoid fever in humans worldwide every year [1]. And *Salmonella enterica* serovar Typhimurium (*S.* Typhimurium) is the most common pathogen of foodborne diseases in areas with poor hygiene and contaminated water resources [2]. It exhibits a wide host range including species phylogenetically distant from birds to rodents, primates and humans [3].

As a clinically important serovar of *Salmonella enterica*, *S.* Typhimurium harbors a virulent plasmid with a highly conserved 8 kb region, the *spv* locus, which is responsible for severe symptoms of Salmonellosis [4, 5]. It could restore virulence to plasmid-cured strains in a mouse model, and was important for the onset of systemic infection by inducing apoptosis of epithelial cells and facilitating intracellular bacterial proliferation in macrophages. The *spv* locus consists of five ORF *spvRABCD*. It has been verified that the *spvB* gene is essential for the virulence [6]. The C-terminal polypeptide of SpvB protein possesses ADP-ribosyltransferase activity and results in depolymerization of F-actin filaments in infected cells [7–9]. It had been reported that SpvB protein was secreted to the cytoplasm of host cells as soon as 1 h post infection (p.i.). However, its cytotoxicity manifested by host cell apoptosis occurred at 10-24 h p.i. [10]. It seemed that SpvB might have an additional virulence function at the early stage of intracellular infection.

Autophagy is an evolutionarily conserved process, which plays a key role in immune defense [11–12]. *Salmonella* infection is considered to be an ideal model to study the mechanisms of autophagy in host immune defense both *in vitro* and *in vivo* [13–14]. It has been reported that autophagy could limit the replication of *S.* Typhimurium in several cell culture models, such as HeLa cells, macrophages and mouse embryonic fibroblasts [15–18]. While *S.* Typhimurium has evolved strategies to subvert the host defense system. Recent studies had revealed that *S.* Typhimurium could interfere with host cell vesicle trafficking, resulting in bacteria escaping from degradation through autophagy [19]. While more exact mechanisms remain to be determined.

Our previous studies had shown that *Salmonella* virulent plasmid harboring *spv* genes could enhance bacterial cytotoxicity by inhibiting autophagy of host cells [20]. It was known that SpvB played an important role in *Salmonella* pathogenesis by depolymerizing of host cell actin, and polymerization of actin cytoskeleton participates in the early events of autophagosome formation [8, 21]. Thus we speculated *spvB* was responsible for the suppression of host cell autophagy by depolymerizing actin. Previous studies had confirmed that the invasion of *S.* Typhimurium with *spv* genes could induce pyroptosis, a form of programmed cell death associated with inflammation [22]. Inflammatory response is important for host against infection, while over-activation of inflammation can lead to cell damage. Autophagy plays a pivotal role in the process of inflammation by regulating a number of cytokines production. Kleinnijenhuis *et al.* reported that deficiency in autophagy led to increased secretion of IL-1 family cytokines in response to *Mycobacterium tuberculosis* infection [23]. However, few studies have been done on the correlation between autophagy and inflammatory response in *Salmonella* infection.

As typical facultative intracellular bacteria, *Salmonella* could cross the epithelial cells layer of small intestine, replicate in phagocytes and disseminate, resulting in systemic infection. In this study, human epithelial HeLa cells, Murine macrophage-like cells J774A.1, and BALB/c mice infected with *S*. Typhimurium carrying or free *spvB* were used *in vitro* and *in vivo* assay, respectively. And SpvB ectopic eukaryotic expression system, as well as cells deficient in essential autophagy components by siRNA were also generated. Our study demonstrated the function of *spvB* in autophagy pathway and the underlying mechanisms.

## RESULTS

### *spvB* inhibited autophagic activity of *S.* Typhimurium infected cells and enhanced intracellular bacterial survival

**Figure 1:**
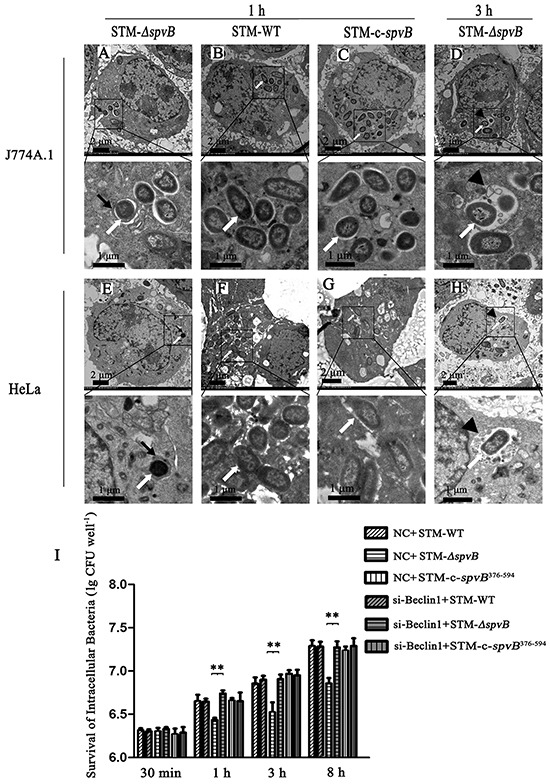
*spvB* inhibited autophagy activity of cells infected with *S.* Typhimurium and enhanced intracellular bacterial survival Ultrastructural features of *S.* Typhimurium infected cells under electron microscope **A-H.** J774A.1 and HeLa cells were infected with STM-*ΔspvB* (A, D, E & H), STM-WT (B & F) and STM-c-*spvB* (C & G) respectively. *S.* Typhimurium infected cells were analyzed at 1 h p.i. (A, B, C, E, F & G) and 3 h p.i. (D & H). Boxed areas were magnified in panels below. Black arrows, autophagosomes; arrowheads, autolysosomes; white arrows, intracellular bacilli. The images were representative of three independent experiments. Assessment of intracellular bacterial survival in J774A.1 cells (**I**). The X axis represented hours p.i. and the Y axis represented lg (CFU well^−1^) (**P <0.01;). 1.5×10^6^ per well infected cells were washed three times with PBS, lysed with 1 ml of 0.2% Triton X-100 for 10 min at the indicated time points (30 min, 1 h, 3 h and 8 h p.i.). The lysates were diluted and plated onto LB agar to enumerate colony-forming units. The data were presented as the mean±S.D. S.D. was calculated from experiments performed in triplicate.

To investigate the effects of *spvB* on autophagy of host cells, J774A.1 and HeLa cells were infected with *S.* Typhimurium wild type strain (STM-WT), *spvB* mutant strain (STM-*ΔspvB*), or complemented strain of STM-*ΔspvB* (STM-c-*spvB*). Ultrastructural features of infected cells were examined by the transmission electron microscopy. Detailed analyses revealed that a double-membrane structure containing undigested bacteria, a characteristic structure of autophagosomes, appeared inside of STM-*ΔspvB* infected cells at 1 h p.i., both in J774A.1 and HeLa cells (Figure 1A & 1E). The autophagosomes subsequently fused with lysosomes, and the single-membrane autolysosomes containing STM-*ΔspvB* and partially digested cytoplasmic materials were observed at 3 h p.i. (Figure 1D & 1H). The double-membrane autophagosomes were absent from cells infected with STM-WT and STM-c-*spvB*. Instead, there were more intracellular bacteria in STM-WT (Figure 1B & 1F), or STM-c-*spvB* infected cells (Figure 1C & 1G).

There was no significant difference in intracellular bacterial survival at 30 min p.i., however, the number of intracellular bacteria were higher in STM-WT and STM-c-*spvB*^376-594^ infected cells than in STM-*ΔspvB* infected cells at 1 h p.i., and the trend maintained during the whole 8 h infection process (Figure 1I). Moreover, inhibition of autophagy pathway with siRNA targeting Beclin 1 (Figure 1I), as well as knockdown of Atg 5 (data not shown), could significantly increase the intracellular bacterial survival in J774A.1 cells.

**Figure 2:**
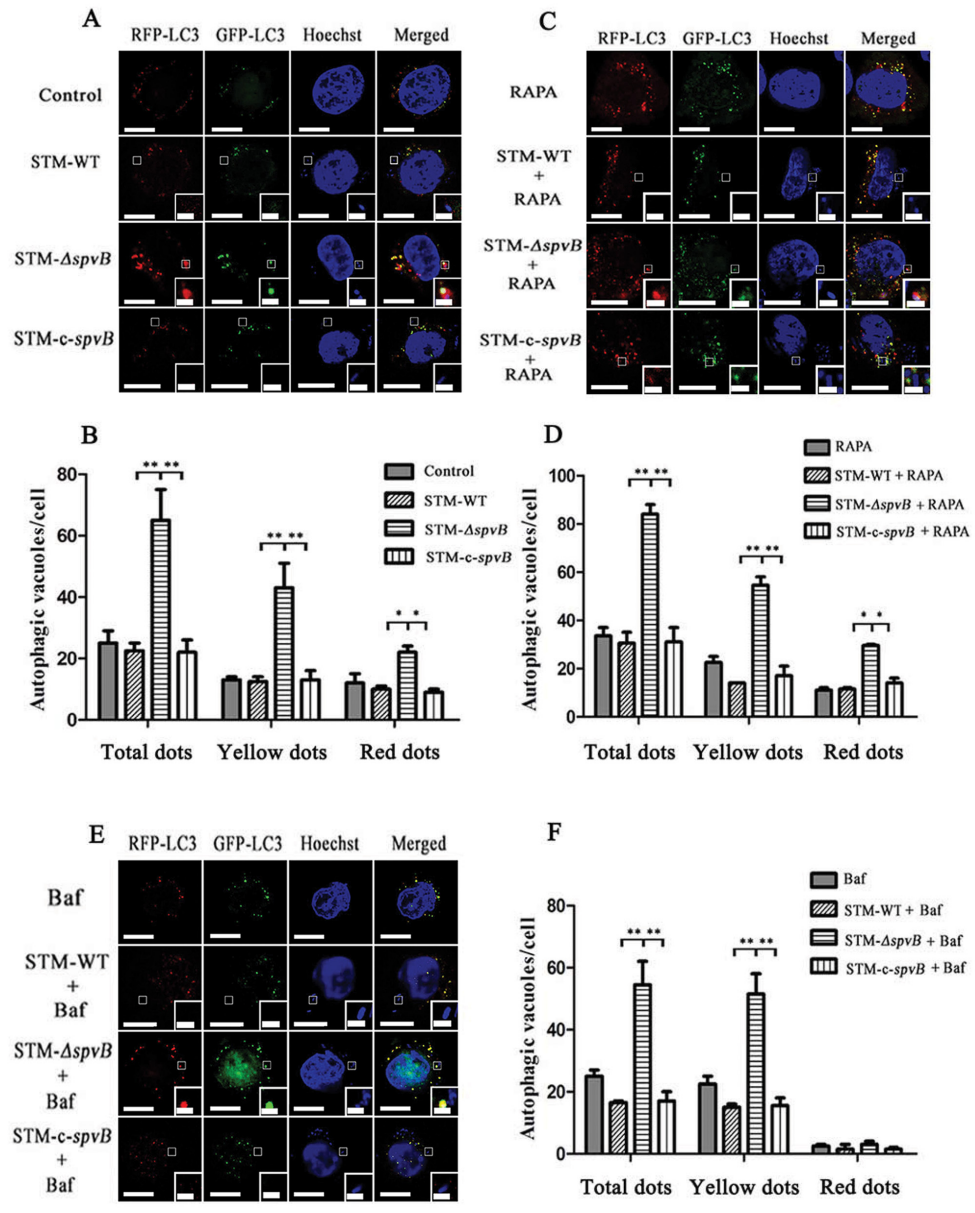
Assessment of punctate LC3 dots in infected HeLa cells HeLa cells stably expressing mRFP-GFP-LC3 were cultured in complete media **A & B.** or media with RAPA **C & D.** for 1 h before infection, or media with Baf for 2 h before infection **E & F.** Bars represented 5 μm or 1 μm in enlarged inset. Number of punctate dots was enumerated in at least 100 cells at 1 h p.i. (**P <0.01; *P < 0.05). The X axis represented the average number of puncta in 100 cells. The data represented as the mean ±S.D. S.D. was calculated from experiments performed in triplicate.

Since autophagy is a dynamic process, the accumulation of autophagosomes in STM-*ΔspvB* infected cells may be due to the increase of autophagosome formation or the inhibition in autophagosomal maturation. Autophagic flux was morphologically traced to distinguish between these two possibilities in this study. mRFP-GFP-LC3 is an autophagic flux indicator, since it shows yellow color (red and green colors merged) in autophagosomes and red color in autolysosomes, due to the quenching of GFP signal by acidic lysosomal pH in autolysosomes, while RFP signal remains stable in acidic pH. HeLa cells stably expressing mRFP-GFP-LC3 were infected with different *S.* Typhimurium strains. It was observed that STM-*ΔspvB* infected cells had visible punctate LC3 structures at 1 h p.i., especially showed as the yellow dots, while STM-WT and STM-c-*spvB* infected cells showed less LC3 puncta (Figure 2A & 2B). When cells expressing mRFP-GFP-LC3 were subjected to Rapamycin (RAPA) treatment to induce autophagy before infection with *S.* Typhimurium, the number of both yellow (autophagosomes) and red (autolysosomes) puncta in STM-*ΔspvB* infected cells was significantly increased (Figure 2C & 2D). However, when Bafilomycin A_1_ (Baf) was used to inhibit the fusion of autophagosome with lysosome, most puncta in STM-*ΔspvB* infected cells are yellow (autophagosome) without a concomitant increase in red puncta (Figure 2E & 2F). Similar trend of the number of punctate LC3 structures was observed in J774A.1 cells transiently transfected with mRFP-GFP-LC3 among groups (Supplementary Figure S1). These results demonstrated that *spvB* could inhibit the formation of autophagosome.

**Figure 3:**
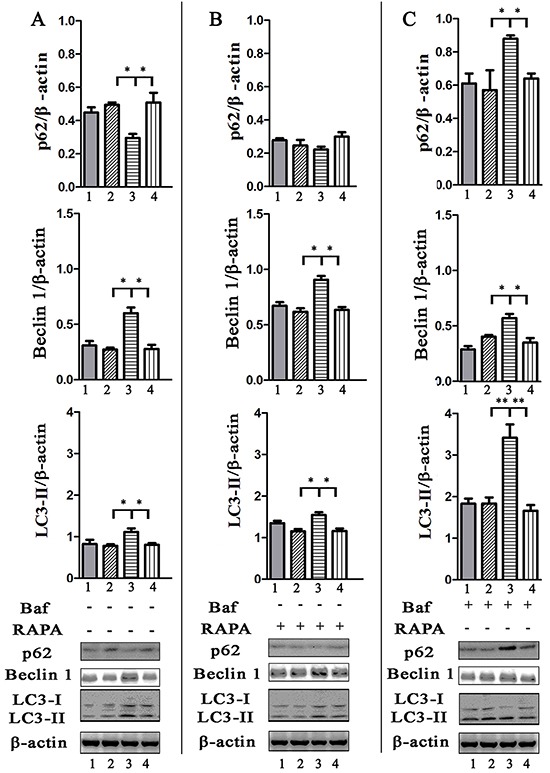
Evaluation of p62, Beclin 1 amount and LC3 turnover in infected HeLa cells by western blotting analysis Cells uninfected (1), or infected with STM-WT (2), STM-*ΔspvB* (3), or STM-c-*spvB* (4) treated with or without RAPA or Baf for 1 h were collected and subjected to western blotting analysis. Semi-quantitative analyses of protein levels based on the density of bands (**P <0.01; *P < 0.05). The results were representative of at least three independent experiments. The data were presented as the mean±S.D. S.D. was calculated from experiments performed in triplicate.

**Figure 4:**
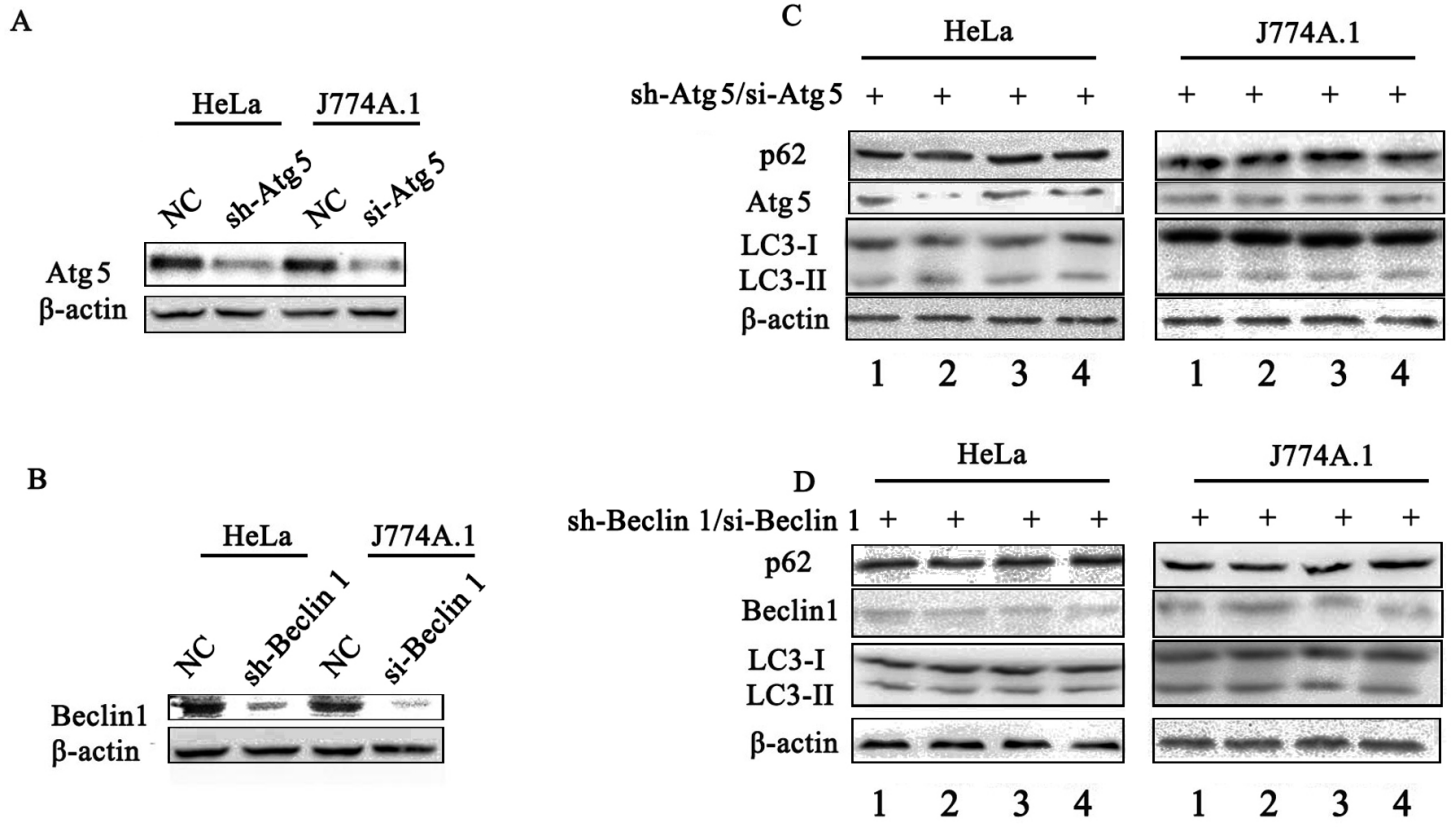
Assay of *S.* Typhimurium infected cells that deficient in essential autophagy components by siRNA Atg 5 **A & C.** and Beclin 1 **B & D.** target siRNA/shRNA was transiently transfected in HeLa cells (shRNA) or J774A.1 cells (siRNA). The level of autophagy protein was detected by western blotting analysis. NC represented the negative control random siRNA.

p62/SQSTM1 is considered as a selective substrate of autophagy by directly binding LC3 and ubiquitin-coated *Salmonella*, and its level inversely correlates with autophagic activity [24]. We found that the level of p62 both in HeLa (Figure 3A) and J774A.1 (Supplementary Figure S2A) cells infected with STM-WT, or STM-c-*spvB* was significantly higher than that in STM-*ΔspvB* infected cells at 1 h p.i. Consistent with the level of p62 protein, both LC3 turnover and Beclin 1 expression increased in STM-*ΔspvB* infected cells, and the treatment of RAPA increased LC3 turnover and Beclin 1 expression in all infected groups (Figure 3B & S2B), while Baf significantly increased the accumulation of p62 in cells infected with STM-*ΔspvB* (Figure 3C & S2C). Meanwhile, suppression the autophagic flux by specifically knockdown of Beclin 1 or Atg 5, which involved in the early stage of autophagosome formation, resulted in a decrease level of both LC3 turnover and Beclin 1 expression in STM-*ΔspvB* infected cells. As a result, there was no significant difference between STM-WT and STM-*ΔspvB* infected cells (Figure 4). These results indicated that *spvB* inhibited host cell autophagy at the stage of autophagosome formation, both in epithelial cells and macrophages.

**Figure 5:**
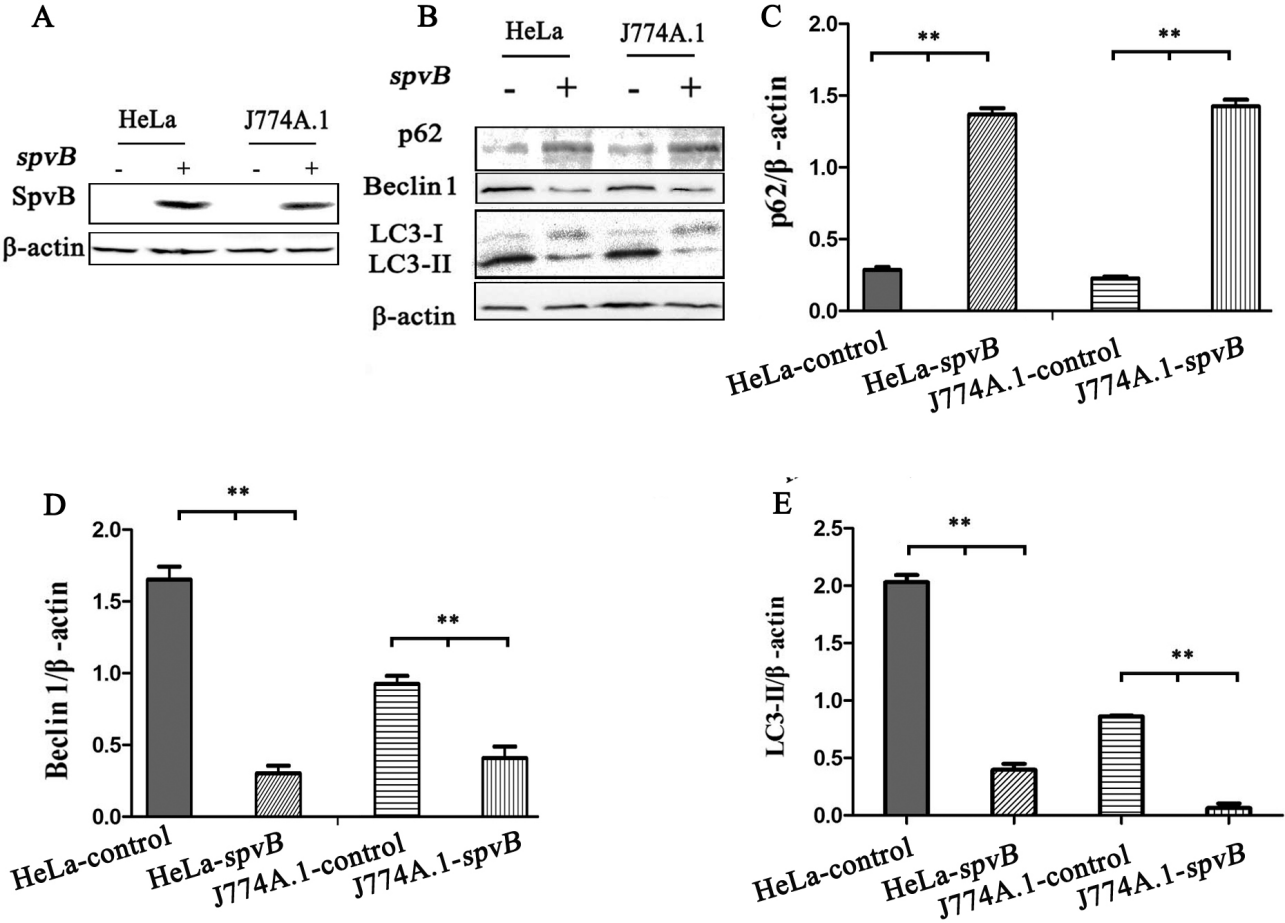
*spvB* overexpression inhibited autophagy activity of host cell HeLa cells and J774A.1 cells transiently expressing *spvB* were detected by western blotting analysis **A.** The level of autophagy protein in *spvB* overexpression cells was analyzed **B.** Semi-quantitative analyses of protein levels based on the density of bands **C, D & E.** (**P <0.01; *P < 0.05). The results were representative of at least three independent experiments. The data were presented as the mean ± S.D. S.D. was calculated from experiments performed in triplicate.

To further investigate whether the inhibition of autophagosome formation is directly due to the effect of SpvB, SpvB was ectopic expressed using pEGFP-N1-SpvB plasmid both in HeLa cells and J774A.1 cells (Figure 5A). Western blotting analysis indicated that both LC3 turnover and Beclin 1 expression decreased in cells transfected with SpvB, while p62 accumulation was enhanced (Figure 5B-5E). These results further demonstrated that *spvB* coud directly inhibit autophagy.

### *spvB* interfered with the initial stage of autophagy by depolymerization of actin cytoskeleton

**Figure 6:**
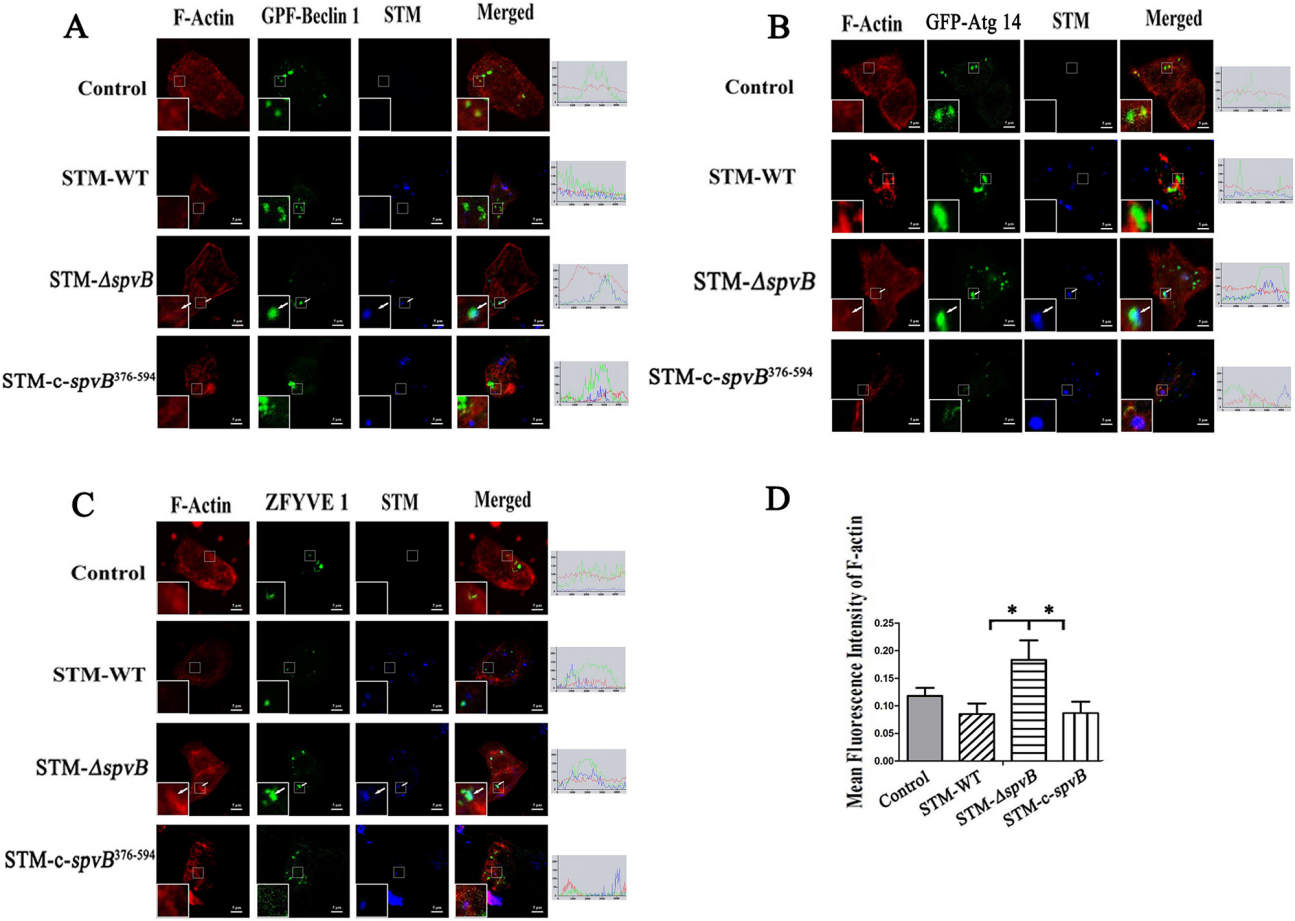
*spvB* interfered with the initial stage of autophagy by depolymerization of actin cytoskeleton HeLa cells transiently expressing EGFP-Beclin 1 **A.** EGFP-Atg 14 **B.** or EGFP-ZFYVE 1 **C.** were infected with *S.* Typhimurium. Arrows denoted the intracellular bacilli (blue) colocalization with protein (green) and actin filaments (red). The images were representative of three independent experiments. Image quantification of F-actin levels was measured by Image J software program in 5 cells **D.** (*P < 0.05). The data were presented as the mean ± S.D. S.D. was calculated from experiments performed in triplicate.

It was reported that actin cytoskeleton participated in autophagosome formation at the early stage [21]. In this study, the colocalizations of Beclin 1 and Atg 14, key members of class III phosphatidylinositol 3-kinase (PI3K-III) complex functioning in the early stage of autophagosome formation, with the actin cytoskeleton and intracellular bacteria were observed. The product of PI3K-III, phosphatidyl inositol 3 phosphate (PtdIns3P) was also probed with a ZFYVE 1. Strikingly, abundant intact cytoskeleton was observed in HeLa cells infected with STM-*ΔspvB*. In these cells GFP-Beclin 1, GFP-Atg 14 and GFP-ZFYVE 1, was frequently colocalized with actin fibers (Figure 6). A few of the intracellular STM-*ΔspvB* bacteria were colocalized with actin fibers. Fewer F-actin fibers were observed in STM-WT infected cells (Figure 6A-6D). As a result, more diffused fluorescent protein was observed in these cells. Although more intracellular bacteria were observed in cells infected with STM-WT at 1 h p.i., the intracellular bacteria were sporadically colocalized with fluorescent protein. The same effects on ZFYVE 1, Beclin 1 and Atg 14 could be observed by using Cytochalasin B (30 μM, Sigma) in cells infected with STM-*ΔspvB* (data not shown), which indicated the inhibition of actin polymerization by SpvB is responsible for the changes in autophagy.

Cells infected with STM-c-*spvB*^376-594^, STM-*ΔspvB* complemented with C-terminal polypeptide of SpvB that possessed ADP-ribosyltransferase activity showed the same trend as STM-WT infected cells. These results suggested that C-terminal polypeptide of SpvB could account for the changes in autophagy (Figure 6). Western blotting analysis also indicated that both LC3 turnover and Beclin 1 expression level was much less in STM-c-*spvB*^376-594^ infected cells compared with STM-*ΔspvB* infected cells (Supplementary Figure S3). These results demonstrated that complemented C-terminal polypeptide of SpvB could restore the inhibtion of host cell autophagic activity.

Western blotting analysis indicated that the level of endogenous Beclin 1 increased in STM-*ΔspvB* infected cells (Figure 3 & Supplementary Figure S2). In line with this, STM-c-*spvB*^376-594^ could restore the suppression of Beclin 1 level (Supplementary Figure S3). PI3K-III complex and its production could provide a platform for autophagosomes formation. These results suggested that *spvB* interfered with the early stage of *S.* Typhimurium-selective autophagy by depolymerization of actin cytoskeleton.

### *spvB* exacerbated inflammatory injury via its suppression of autophagy

**Figure 7:**
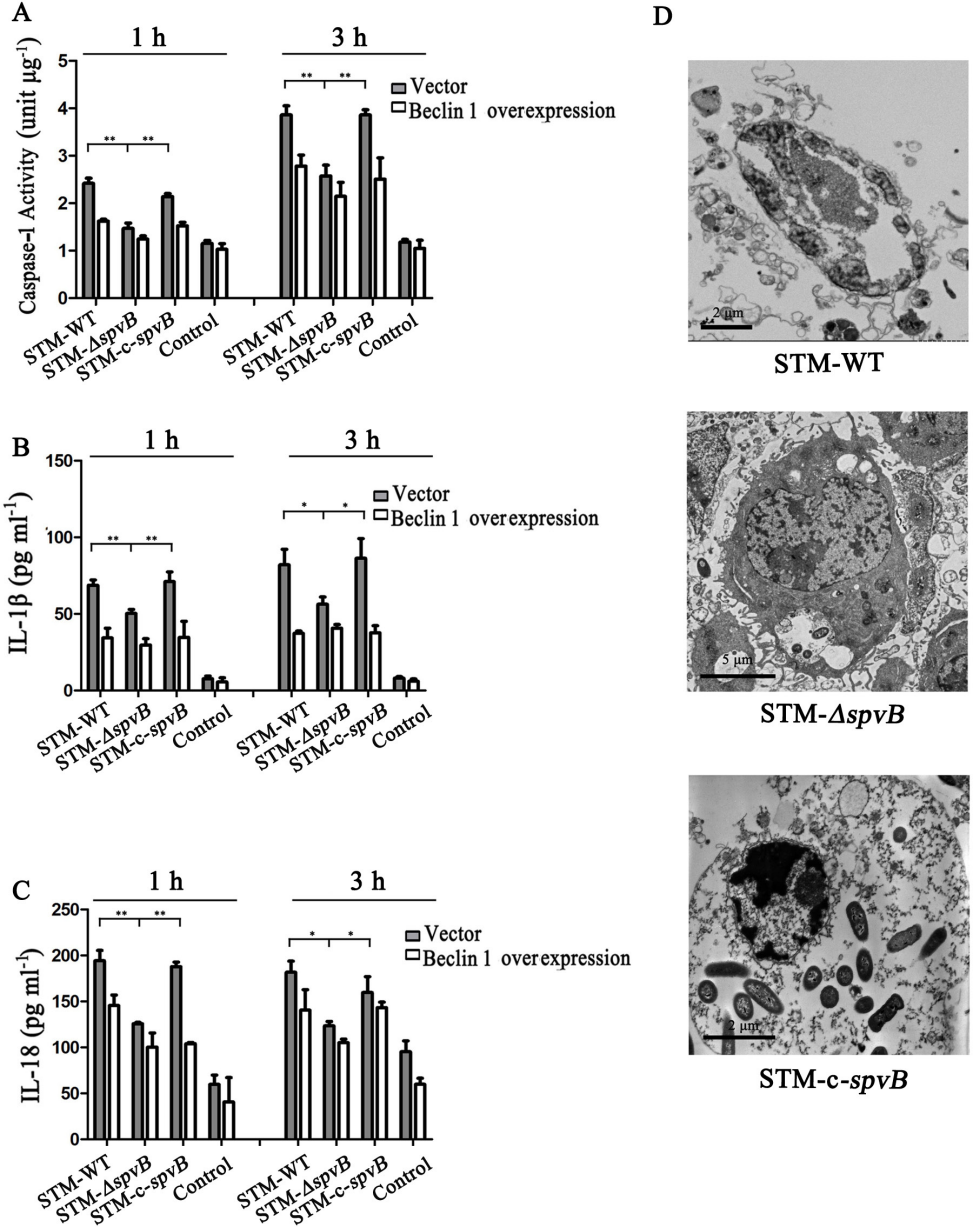
*spvB* resulted in pyroptosis of J774A. 1 cells Caspase-1 activities **A.** IL-1β **B.** and IL-18 **C.** in supernatants of infected J774A.1 cells transiently tranfected with empty vector and Beclin 1 (**P <0.01; *P < 0.05). The data were presented as the means ± S.D. S.D. was calculated from experiments performed in triplicate. Pyroptosis in J774A.1 cells infected with STM-WT & STM-c-*spvB* at 3 h p.i. **D.**

It was reported that *Salmonella* infection stimulated pyroptosis of macrophages, a newly identified program of caspase-1-correlated cellular demise, resulting in cell lysis with release of IL-1β and IL-18 [22, 25]. We found that caspase-1 activity of J774A.1 cells was increased at 1 h and 3 h after infection with all three *S.* Typhimurium strains. Nevertheless, compared with STM-*ΔspvB* infected group, caspase-1 activity was much higher in STM-WT, or STM-c-*spvB* infected groups (Figure 7A). To investigate whether the increased activity of caspase-1 in *spvB* positive strain infected cells were due to the suppression of autophagy, exogenous Beclin 1 were overexpressed in J774A.1 cells. It was demonstrated that caspase-1 activity decreased in Beclin 1 overexpressed cells, resulting in no significant difference among groups. The secretion of IL-1β and IL-18 displayed the same trend as caspase-1 (Figure 7B & 7C). Meanwhile, morphological features of pyroptosis, pore formation in the cell membrane, nuclear condensation, cell swelling and bursting, were observed in STM-WT and STM-c-*spvB* infected J774A.1 cells under the transmission electron microscope (Figure 7D).

To further investigate the effects of *spvB* on autophagy of host cells *in vivo*, BALB/c mice were infected i.p. with 200 CFU of STM-WT or STM-*ΔspvB*. The peritoneal macrophages were collected at 1 d p.i. and analyzed by western blotting. Consistent with the results of experiments *in vitro*, turnover of LC3-I to LC3-II and the expression of Beclin 1 were also increased in peritoneal macrophages of STM-*ΔspvB* infected mice compared with STM-WT infected mice, indicating that *spvB* also suppressed host cell autophagy *in vivo* (Supplementary Figure S4A-S4C). We further determined whether the suppression of autophagy had an influence on the spreading of bacteria *in vivo*. As shown in Supplementary Figure S4D, the number of bacteria in livers was higher in STM-WT infected mice at 4 d p.i. Bacteria persisted throughout a 7 d time-course. Proinflammatory cytokines, IL-1β and IL-18, increased in the sera of STM-WT infected mice at 1 d p.i. With the prolongation of infection, concentration of the two cytokines in the sera of STM-WT infected mice decreased, while they were still higher than those in STM-*ΔspvB* infected mice (Supplementary Figure S4E & S4F).

**Figure 8:**
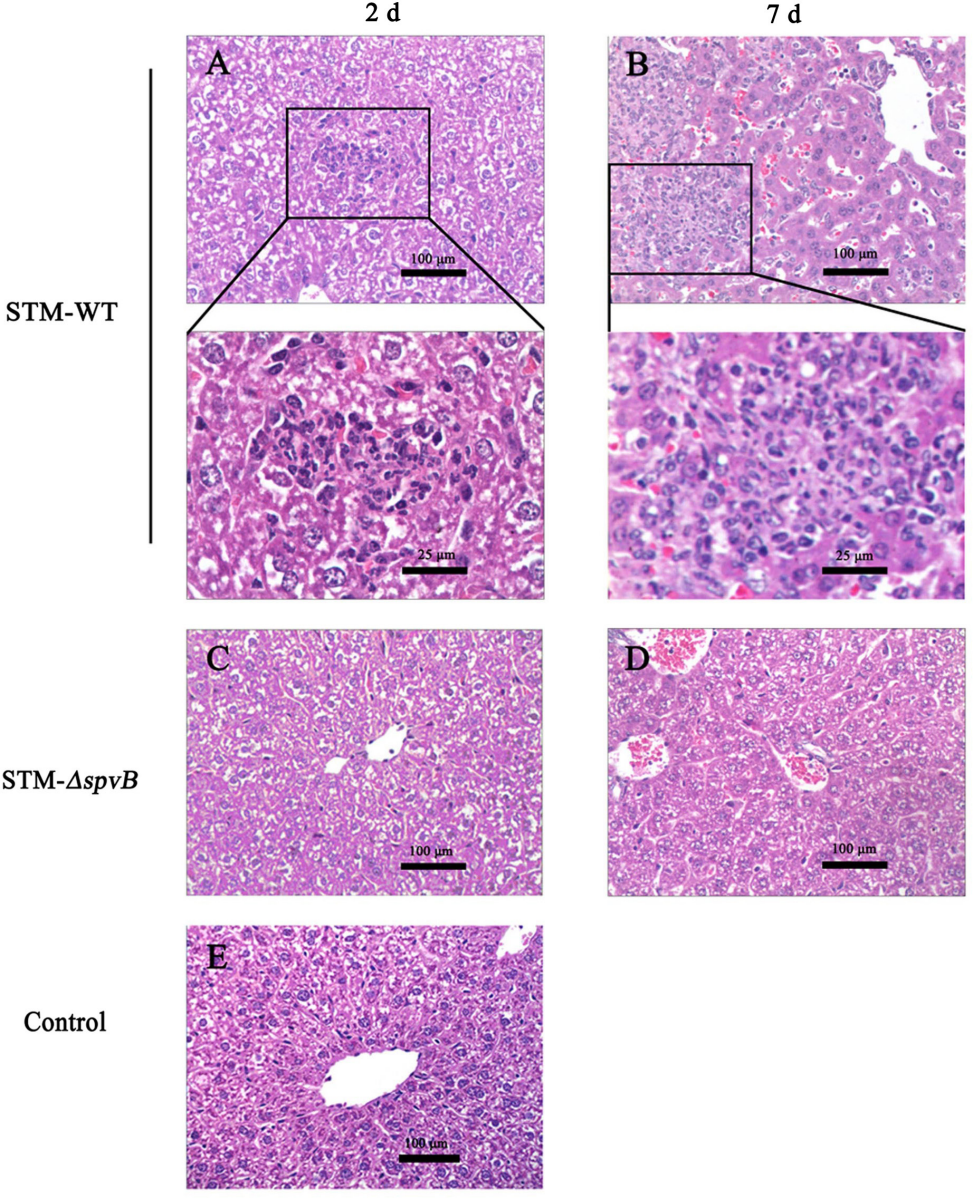
Histopathologic analysis of the infected murine livers Livers of mice infected with STM-WT showed infiltration of inflammatory cells at 2 d p.i **A.** and aggravated at 7 d p.i. **B.** Boxed areas were magnified in panels below. While the histopathological changes of mice infected with STM-*ΔspvB* were alleviated **C & D.** Histology of murine liver from control group was shown in **E.**

At 2 d p.i., livers of mice infected with STM-WT showed significant pathological changes including cell swelling, focal necrosis, and inflammatory cell infiltration. The lesions aggravated at 7 d p.i. While the histopathological changes of mice infected with STM-*ΔspvB* were slight, and there were no significant differences between infected mice and control mice at 2 d p.i., and the lesions were alleviated compared with STM-WT infected mice at 7 d p.i. (Figure 8).

## DISCUSSION

Autophagy is a fundamental cell biology process which maintains the cellular homeostasis by degradation of damaged organelles, redundant proteins and invading pathogens. Cells rely on autophagy to carry out the basal housekeeping role in danger signal removal and self repair [24]. Immune cells can eliminate intracellular bacteria, present antigens and regulate cytokines secretion by autophagy [26–28]. In line with this, certain pathogens have evolved some mechanisms to evade autophagy. *Salmonella* infection has gained recognition as a paradigm for autophagy [13, 29]. Recently, Tattoli *et al.* found that *S.* Typhimurium escaped from autophagy by an unknown mechanism [19]. Our previous study demonstrated that the *Salmonella* virulence plasmid harboring *spv* genes could enhance intracellular bacterial growth by suppressing autophagy of host cells [20]. However, the exact mechanisms were not yet elucidated. Here, we suggested for the first time that *spvB* could inhibit autophagy of host cells for *Salmonella* benefits.

Autophagosomes and autolysosomes specifically enwrapping intracellular bacteria were observed in STM-*ΔspvB* infected cells under electron microscope, indicating that *spvB* was involved in inhibiting host cell autophagic activity. As autophagy is a dynamic process, two possibilities can be applied to explain for fewer autophagic structures in STM-WT infected cells, inhibition of autophagosome formation or rapid maturation to autolysosomes. Autophagic flux is considered as a reliable indicator for cellular autophagic activity. The degradation of specific substrates and LC3 turnover were regarded as the reliable approaches to assess autophagic flux [24, 26]. To fully understand the process, pharmacological approaches are usually employed to block specific stages of autophagy. In this study Baf, which inhibits autophagosome-lysosome fusion to block the substrate degradation, is employed in assessment of autophagic flux. We found p62 levels in host cells infected with STM-WT and STM-c-*spvB* were significantly higher comparing with those in STM-*ΔspvB* infected cells. This indicated that the autophagic flux in STM-WT and STM-c-*spvB* infected cells was decreased, either at the early step of autophagosome formation or the later step of autolysosome degradation. Significant accumulation of p62 was observed among all infected groups treated with Baf. On the contrary, STM-*ΔspvB* induced the turnover of LC3-II in infected cells. As described above, decreasing level of LC3-II might be resulted from the inhibition of autophagosome formation or the highly activated autophagic flux of degradation process. Based on the results of p62 levels and LC3-II turnover, we concluded that *spvB* is involved in inhibiting autophagosome formation. Consistent with this, morphologically tracked autophagosomes (red puncta) and autolysosomes (yellow puncta) with mRFP-GFP-LC3 tandem construct indicated that bacteria harboring *spvB* inhibited the formation of autophagosomes. As infection is complicated, lots of bacteria factors involves in this process. To further explore the direct evidence for *spvB* inhibition autophagy, SpvB were ectopic expressed both in epithelial cells and macrophages. Both LC3 turnover and Beclin 1 expression decreased in cells that ectopic expressed SpvB. It provided the direct evidence that *spvB* could inhibit autophagy. In line with this finding, it had been confirmed that the *Salmonella* virulence plasmid harboring *spv* genes was involved in blocking the formation of autophagosome in the earlier autophagy process in our previous study [20].

PI3K-III complex (composed of Vps34, Vps15, Beclin 1 and Atg 14) is responsible for providing a platform at the stage of autophagosome formation [30–33]. Previous study had demonstrated that both the constituent of PI3K-III complex and its product, PtdIns3P were colocalized with actin filaments [21]. Depolymerization of actin filaments could inhibit the formation of autophagosomes [21]. In this study, we verified *spvB* suppression of autophagic flux was due to its function in depolymerizating F-actin. Since the microfilaments in epithelial cells are abundant, F-actin of HeLa cells was stained with phalloidin-rhodamine in this study. Consistent with previous studies, infection by *spvB* positive strains resulted in a reduction of actin filaments, and more diffused PI3K-III complexes were observed in the cytoplasm. On the contrary, most of the PI3K-III complexes were located on actin cytoskeleton of STM-*ΔspvB* infected cells, and some of the intracellular bacteria were colocalized with the complexes. It has been reported that C-terminal polypeptide of SpvB possesses ADP-ribosyltransferase activity. We found that STM-c-*spvB*^376-594^, STM-*ΔspvB* complemented with C-terminal polypeptide of SpvB, could restore STM-*ΔspvB* with inhibtion of host cell autophagic activity.

Inflammation is a protection process for host in response to infection and tissue damage [21]. Bacteria invasion can induce cytokines secretion of host cells against infection. While over-activation of the inflammation pathways would eventually alter the host cell homeostasis that may lead to tissue damage. It had been reported that *Salmonella* infection could cause pyroptosis of macrophage. In the present study, we found that *spvB* could induce caspase-1 activation in macrophages and increase the secretion of IL-1β and IL-18. Browne *et al*. reported that *spvB* was responsible for the delayed macrophage pyroptosis [5]. However, pyroptosis was observed at 3 h p.i. in this study. The discrepancy may be due to the differences in MOI (1:1 vs. 100:1). Previous studies had demonstrated that blocking autophagy by genetic deletion of Atg 16L1 led to increased cytokines production [34]. However, the exact role of autophagy in regulation of inflammatory responses to *S.* Typhimurium infection remains unclear. In our study, we found that the secretion of IL-1β and IL-18 were significantly decreased when essential autophagy protein Beclin 1 was overexpressed. The results suggested that pyroptosis induced by *spvB* was correlated with the suppression of host cell autophagy. With the stimulation of STM-*ΔspvB* infection, activated autophagy of the host could act as a self-protection mechanism to limit bacterial proliferation and clear bacterial effectors. Meanwhile pro-IL-1β and other proinflammatory cytokines could be sequestered and degraded by autophagy. While the inhibition of autophagy by *spvB* positive strains resulted in the accumulation of inflammatory-activating stimulus, leading to macrophage pyroptosis.

Immune responses against *S.* Typhimurium infection are more complex *in vivo*. To investigate whether *spvB* suppresses autophagy and aggravates infection *in vivo*, BALB/c mice were infected i.p. with *S.* Typhimurium. As expected, autophagic activity of the peritoneal macrophages isolated from STM-WT infected mice was inhibited. The viable bacteria can be isolated from murine livers at 2 d p.i., and the significant difference between STM-WT and STM-*ΔspvB* infected mice appeared at 4 d p.i. The number of bacteria in STM-WT infected mice was much higher than that in STM-*ΔspvB* infected mice. Data suggested that *spvB* could suppress autophagy in macrophages *in vivo* for bacterial growth and dissemination. These results provided a new pathogenic mechanism of SpvB in *S.* Typhimurium systemic infection via suppressing host cell autophagy. To further convince our findings, we cloned *spvB* onto a eukaryotic expression vector and performed some studies in uninfected cells, results ensured *spvB* function in autophagy.

In summary, the present study demonstrated that *spvB* could inhibit autophagosome formation at the early stage of autophagy via depolymerization of F-actin filaments, and aggravate inflammatory injury of the host. These results provide novel insights into the roles of *spvB* in the processes of infection, which has potential application in prevention and treatment of *Salmonella* infection.

## MATERIALS AND METHODS

### Bacterial strains and cell culture

*S.* Typhimurium strains used here included STM-WT, STM-*ΔspvB*, STM-c-*spvB* and STM-c-*spvB*^376-594^. Wild type *S.* Typhimurium was kindly provided by Professor Roy Curtiss III [9]. *spvB*-mutant strain STM-*ΔspvB* was constructed by suicide plasmid PGMB151 homologous recombination with STM-WT. STM-c-*spvB* was complemented STM-*ΔspvB* with plasmid pBAD encoding *SpvB*. STM-c-*spvB*^376-594^ was complemented STM-*ΔspvB* with plasmid pBAD encoding C-terminal polypeptide of SpvB (amino acids 376-594). All bacterial strains were grown in Luria-Bertani (LB) broth at 37°C. Murine macrophage-like cell line J774A.1 and human epithelial HeLa cell line (ATCC) were cultured in RPMI 1640 medium (Sigma) supplemented with 10% fetal calf serum (HyClone). Cells were cultured in a humidified incubator containing 5% CO_2_ and 95% free air at 37°C. HeLa cells stably expressing mRFP-GFP-LC3 were constructed in this study. The mRFP-GFP-LC3 tandem construct was a gift from Tamotsu Yoshimori (Osaka University) [35]. For construction of the *spvB* overexpression plasmid, full length of *spvB* gene was inserted into pEGFP-N1. Lipofectamine 2000 (Invitrogen) was used for transfection according to the manufacturer's instructions. G418 (600 ng μl^−1^, Invitrogen) was added 48 h post transfection to select drug-resistant cells, and cloning of positive cells was performed by serial dilution. HeLa cells stably expressing mRFP-GFP-LC3 were maintained in complete medium containing G418 (300 ng μl^−1^).

### RNA interference of Beclin 1 and Atg 5

Cells were seeded in plates and incubated overnight. HeLa cells were transfected with negative control shRNA, Beclin 1 target-, or Atg 5 target- shRNA (provided by Shanghai Genepharma Co,. Ltd) via Lipofectamine 2000. Beclin 1 and Atg 5 of J774A.1 cells were knockdown by small interfering RNA (siRNA). The interfering sequence of Beclin 1 was TTGATTGTGCCAAACTGTC, and sequence of Atg 5 was CGAATTCCAACTTGCTTTA. The negative control random siRNA was provided by Sangon Biotech Co,. Ltd.

### Bacterial infection

Overnight bacterial cultures were diluted 20-fold in LB medium containing 0.3 M NaCl and subcultured in aerobic conditions for 3 h. Before infection, bacteria were quantified spectrophotometrically by determining the optical density at 600 nm along with viable plate counts. Mammalian cells were seeded in plates 16-24 h before infection. The next day cells were washed three times with PBS, incubated with HBSS for 0.5 h, and infected with *Salmonella* at a multiplicity of infection (MOI) of 100:1. Cells were washed with PBS, and fresh medium containing amikacin (100 μg ml^−1^) was added to kill the extracellular bacteria at 1 h p.i. Baf (100 nM, Sigma), a microtubule-disrupting agent, was added to the complete medium 2 h before infection to inhibit autophagosome-lysosome fusion. To activate autophagic activity, cells were pretreated with mTOR inhibitor rapamycin (4 μM, Sigma) for 1 h before infection. At different time points following infection, cells were processed in the following ways.

### Transmission electron microscopy

Infected cells were pelleted by centrifugation, and then fixed with 2.5% glutaraldehyde in 0.1 M phosphate buffer. Samples were post-fixed in 1% osmium tetroxide, and dehydrated through a series of graded acetone washes. Following this, samples were embedded in epoxy resin, sectioned, and stained with uranyl acetate and lead citrate. The sections were subsequently examined under transmission electron microscope (HT7700, Hitachi Co., Japan).

### Plasmid transfection and confocal microscopy

Cells were transiently transfected with plasmid EGFP-Atg 14, EGFP-Beclin 1, Flag-Beclin 1, or pEGFP-ZFYVE 1 by Lipofectamine 2000 according to the manufacturer's instructions. For immunofluorescence microscopy, bacteria were stained with rabbit anti-*Salmonella* O-Ag serum (TBC) and DyLight 405-conjugated goat anti-rabbit IgG (Beyotime). Transiently transfected cells were infected with *S.* Typhimurium at 48 h after transfection, then fixed with 4% paraformaldehyde in PBS, and stained with Phalloidin-Rhodamine and/or DAPI. The samples were imaged under a confocal laser scanner microscope (Fluoview FV1000, Olympus, Japan). F-actin levels were measured by Image J software program. For quantification of punctate LC3 structures, GFP-LC3 and mRFP-LC3 punctate dots were counted in more than 100 cells.

### Western blotting analysis

The polyclonal sheep anti-Beclin 1 antibody and peroxidase conjugated Donkey anti-sheep IgG antibody were purchased from R&D Systems. The monoclonal mouse antibody against MAP1LC3B and polyclonal rabbit antibody against p62/SQSTM1 were purchased from MBL. Peroxidase conjugated goat anti-rabbit IgG and goat anti-mouse IgG antibody were purchased from ABGENT. The rabbit anti-SpvB antibody was obtained by our lab.

Infected cells were collected and lysed in RIPA buffer (50 mM Tris.HCl, 150 mM NaCl, 1% (w v^−1^) NP-40, 5% (w v^−1^) sodium deoxycholate, 0.1% (w v^−1^) SDS, 1 mM EDTA, 1 mM phenylmethylsulfonyl fluoride and 2 μg ml^−1^ leupeptin) on ice for 10 min. Total protein of cell lysates was measured using BCA assay kit (Beyotime) and 45 μg protein from each sample was loaded into each lane. Proteins were separated by 12% SDS-PAGE, followed by transfer onto a nitrocellulose membrane (Pall Corporation). The following membrane blocking, antibody incubations and chemiluminescence (ECL, Biological Industries) detection were performed according to the reference [20]. The amount of correlated proteins was analyzed by Image J launcher broken symmetry software program.

### Assay of caspase-1 activity

After infection with *S.* Typhimurium for 1 h and 3 h, the macrophages were collected. The caspase-1 activity in infected cell lysates was measured using the caspase-1 activity assay kit (Beyotime) and the protein concentrations were determined by the Bradford protein assay kit (Beyotime). One unit of caspase-1 vitality is definded as the amount of enzyme that will cleave 1.0 nmol of the substrate per hour at 37°C under saturated substrate concentration. Relative caspase-1 activity was expressed as the ratio of caspase-1 vitality to the total protein concentration (unit μg^−1^)

### Cytokines assay

The concentration of IL-1β and IL-18 secretion from macrophages during *S.* Typhimurium infection was analyzed by ELISA kits (eBioscience) according to the manufacture's instructions. Cell culture supernatants were collected at 1 h and 3 h p.i, and the concentration of cytokines was calculated using standards and a standard curve.

### Mice experiment

Female BALB/c mice, 6- to 8-week-old, were used in these experiments. All animal were purchased, bred and kept in specific pathogen-free barrier facilities at the experimental animal center of Soochow University.

Mice were randomized into one of the three groups, control, STM-WT and STM-*ΔspvB* infection groups. Mice in the control group were injected with 0.9% saline through intraperitoneal (i.p.); while other groups were infected i.p. with 200 CFU of *S.* Typhimurium strains respectively. At 1, 2, 4 and 7 d p.i., five infected mice from each group were euthanized by cervical dislocation. Macrophages were washed from peritoneal cavities with 10 ml of RPMI 1640 medium (Sigma). After being centrifuged for 15 min, cells were resuspended in RPMI 1640 medium containing 10% fetal calf serum (HyClone). This medium was added to 6-well plates and cultured in a humidified incubator containing 5% CO_2_ and 95% free air at 37°C for 2 h. Then plates were washed in RPMI 1640 medium and the adherent macrophages were collected. Murine livers were aseptically removed from both infected and control mice.

### Ethics statement

All animal experiments were approved by the Animal Experimental Committee of the Soochow University (Grant 2111270) and were in accordance with the National Institutes of Health Guidelines for the Care and Use of Laboratory Animals (NIH Guidelines).

### Histopathologic analysis

Tissue samples of livers from infected mice were fixed in 4% paraformaldehyde at 4°C overnight. Then tissues were processed with routine histological procedures, paraffin embedding, section cutting and deparaffinization. The sections were stained with hematoxylin-eosin and observed under a light-microscope (Olympus, Japan).

### Statistics

All data points represent as the mean ± standard deviation (S.D.) from at least three experiments. Statistical analyses were performed using Student's *t*-test. Significant differences: *P < 0.05; **P < 0.01.

## SUPPLEMENTARY FIGURES


