# Supplementary material for: A novel contribution of spvB to pathogenesis of Salmonella Typhimurium by inhibiting autophagy in host cells
Source: Oncotarget. 2016 Jan 22;7(7):8295–309. doi: 10.18632/oncotarget.6989 (PMC4884993; doi:10.18632/oncotarget.6989)
Supplement: Supplementary file 1 [file oncotarget-07-8295-s001.pdf]

SUPPLEMENTARY FIGURES

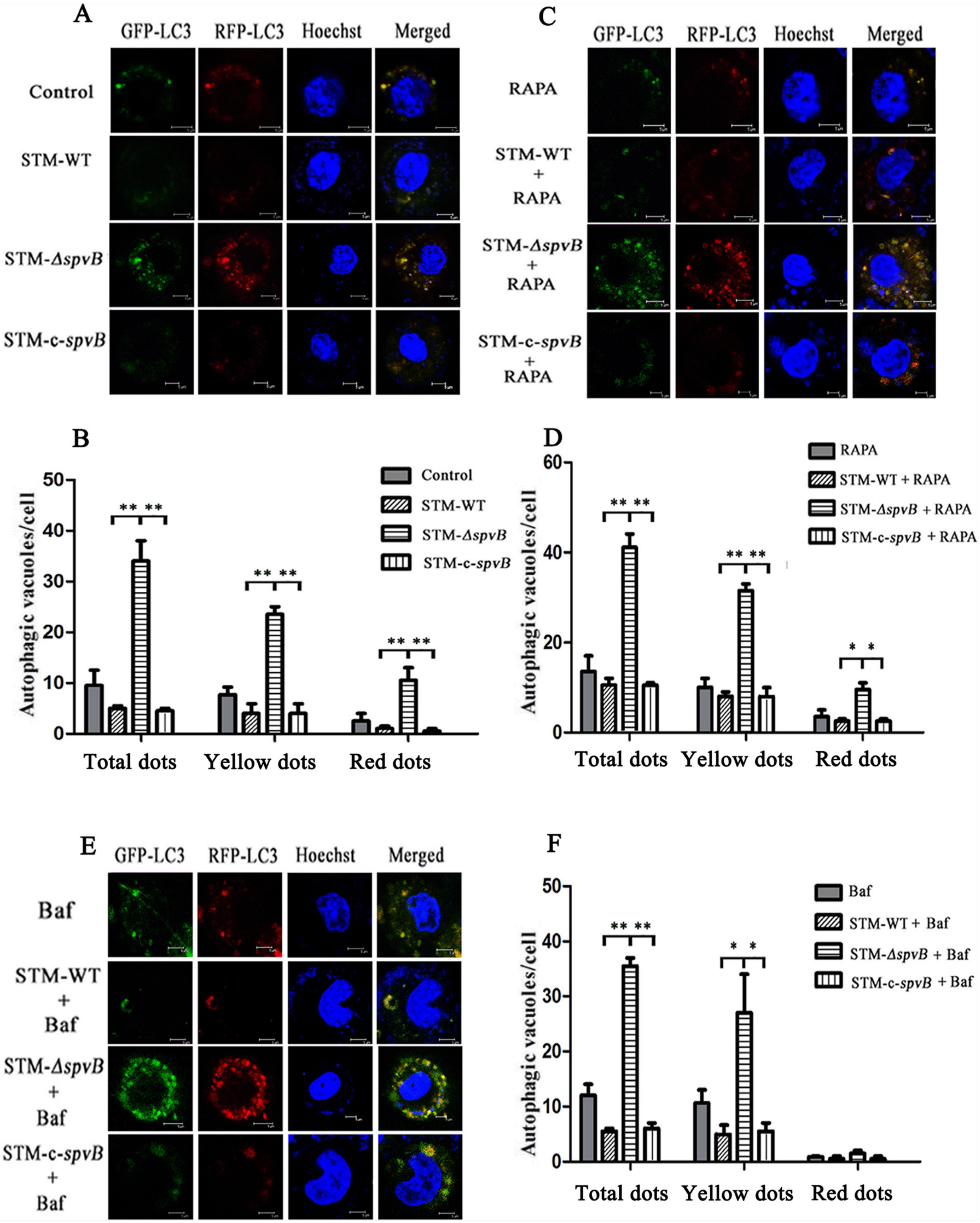

Supplementary Figure S1: Assessment of punctate LC3 dots in infected J774A.1 cells. Cells transiently transfected with mRFP-GFP-LC3 were cultured in complete media **A & B.** or media with RAPA **C & D.** for 1 h before infection, or media with Baf for 2 h before infection **E & F.** Number of punctate dots was enumerated in at least 100 cells at 1 h p.i. (***P* < 0.01; **P* < 0.05). The data represented as the mean ±S.D. S.D. was calculated from experiments performed in triplicate.

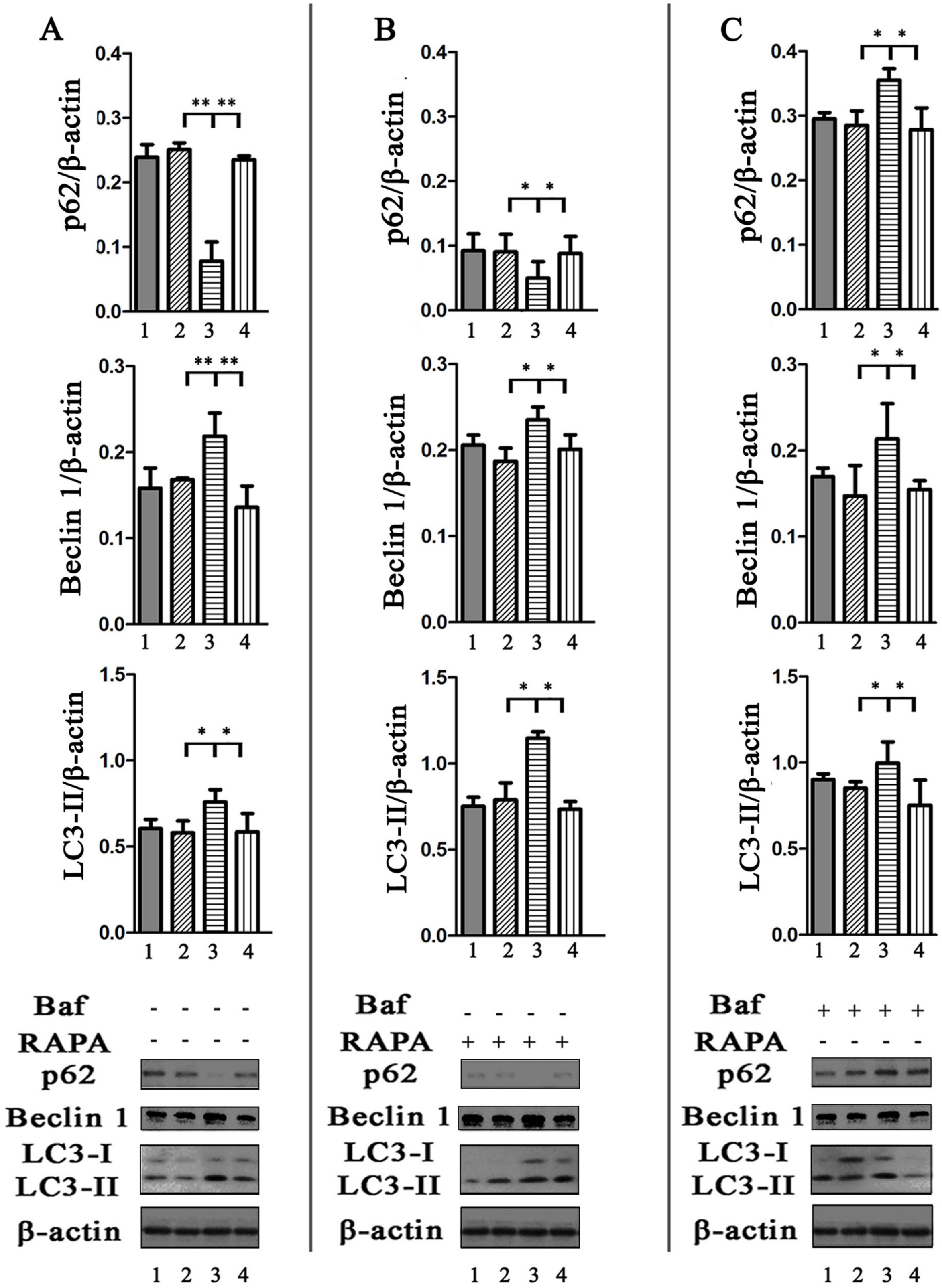

Supplementary Figure S2: Evaluation of p62 and Beclin 1 amount, and LC3 turnover in infected J774A.1 cells by western blotting analysis. Cells uninfected (1), or infected with STM-WT (2), STM-*ΔspvB* (3), or STM-c-*spvB* (4) treated with or without RAPA or Baf for 1 h were collected and subjected to western blotting analysis. Semi-quantitative analyses of protein levels based on the density of bands (***P* < 0.01; **P* < 0.05). The results were representative of at least three independent experiments. The data were presented as the means ± S.D. S.D. was calculated from experiments performed in triplicate.

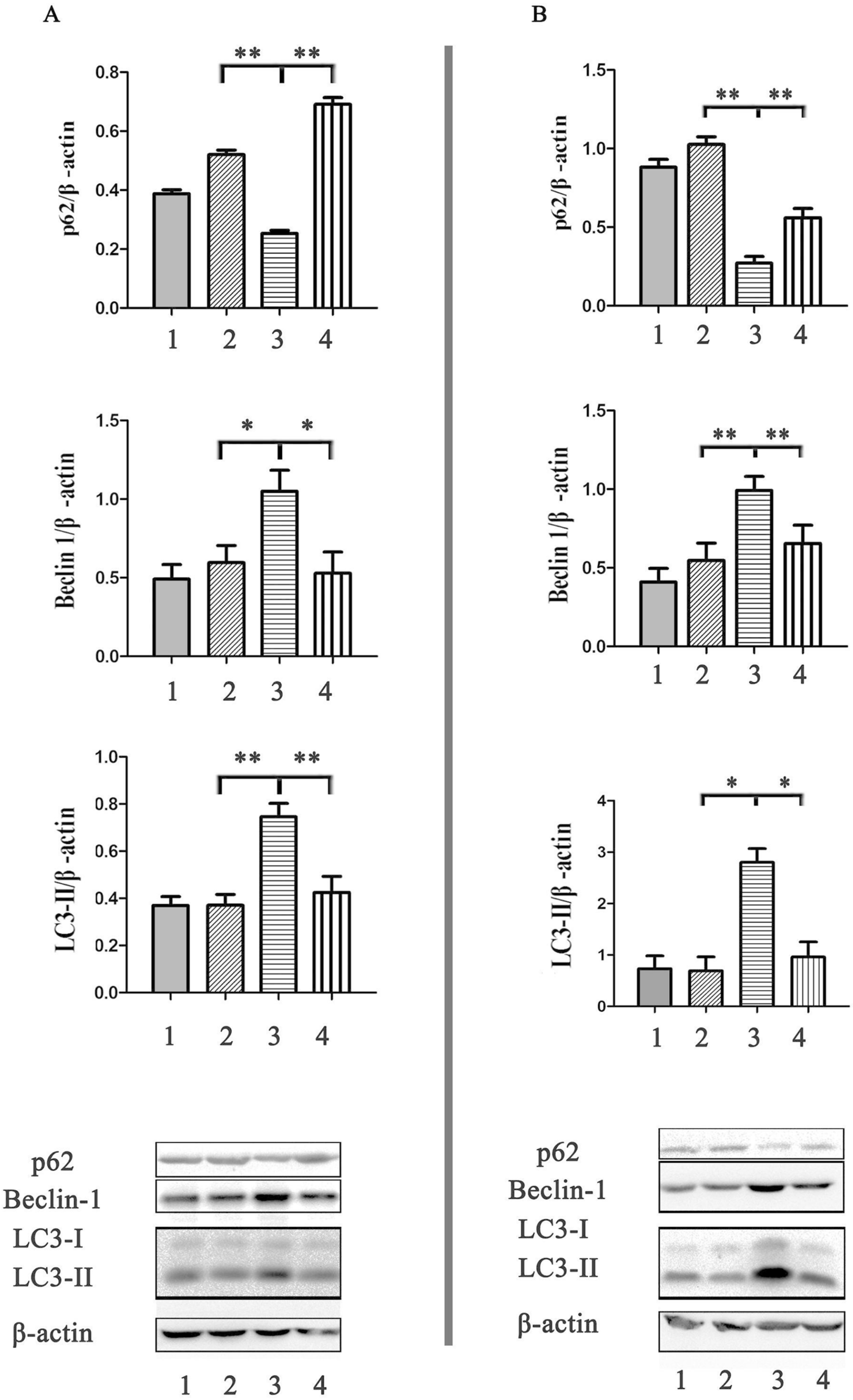

Supplementary Figure S3: Evaluation of autophagic protein expression in STM-c-*spvB*$^{376–594}$ infected cells by western blotting analysis. HeLa cells **A.** or J774A.1 cells **B.** uninfected (1), or infected with STM-WT (2), STM-*ΔspvB* (3), or STM-c-*spvB*$^{376–594}$ (4) were collected and subjected to western blotting analysis. Semi-quantitative analyses of protein levels based on the density of bands (**$P < 0.01$; *$P < 0.05$). The results were representative of at least three independent experiments. The data were presented as the mean ± S.D.. S.D. was calculated from experiments performed in triplicate.

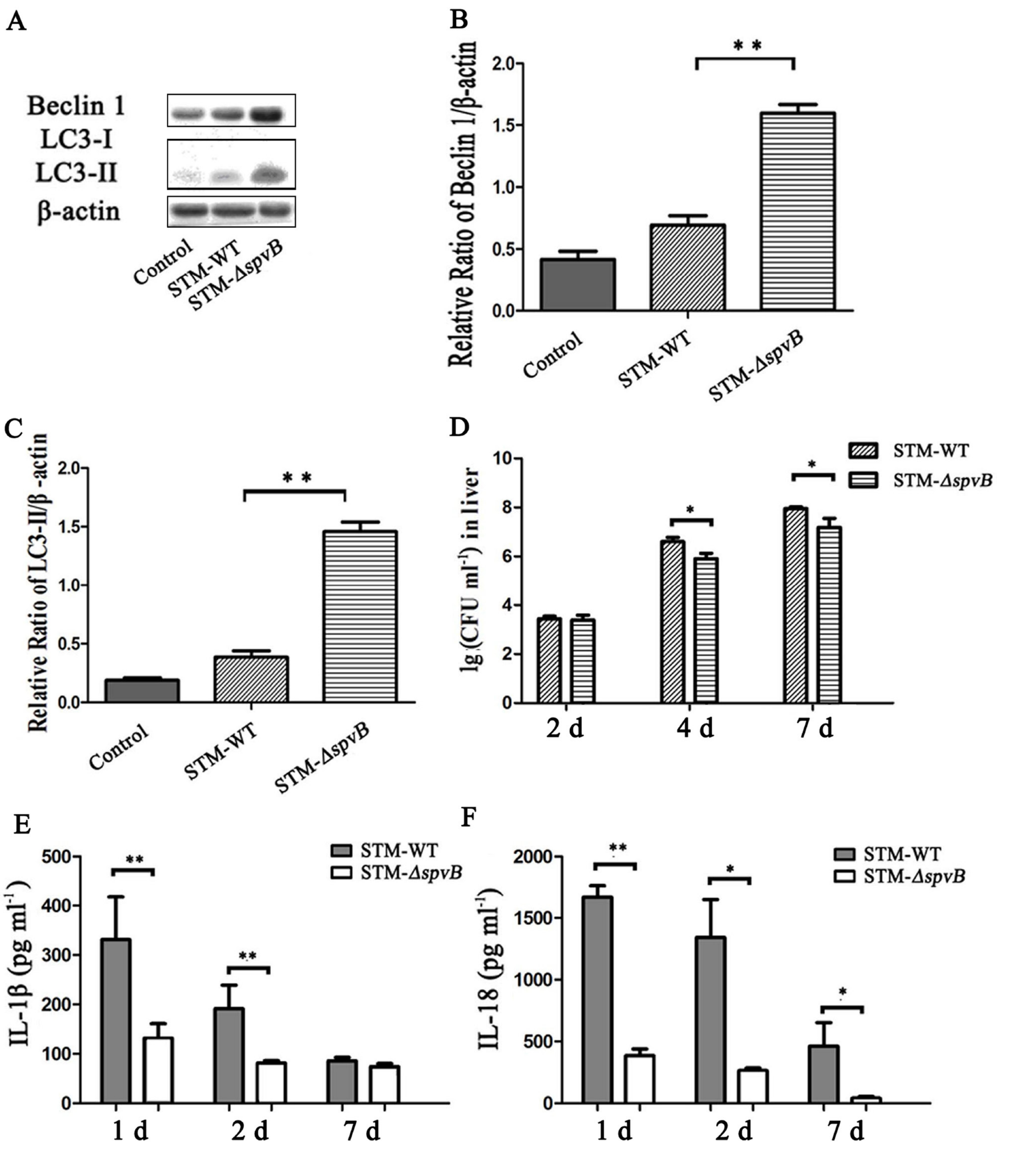

Supplementary Figure S4: *spvB* suppressed macrophages autophagy while increased inflammatory cytokines secretion *in vivo*. Determination of autophagy protein expression in peritoneal macrophages **A, B & C.** Quantitation of the viable bacteria isolated from murine livers **D.** The Y axis represented the logarithm values of bacterial number in livers. (CFU ml^{-1}). Results were given as geometric means for five mice (*$P < 0.05$). IL-1β **E.** and IL-18 **F.** concentration in the sera of mice infected with STM-WT and STM-*ΔspvB* (**$P < 0.01$; *$P < 0.05$).
